# Fabla: A voice-based ecological assessment method for securely collecting spoken responses to researcher questions

**DOI:** 10.3758/s13428-025-02777-1

**Published:** 2025-08-13

**Authors:** Deanna M. Kaplan, Santiago J. Arconada Alvarez, Roman Palitsky, Hyoann Choi, Gari D. Clifford, Melese Crozier, Boadie W. Dunlop, George H. Grant, Morgan N. Greenleaf, Leslie M. Johnson, Jessica Maples-Keller, Holly F. Levin-Aspenson, Jennifer S. Mascaro, Ariel McDowall, Nicole S. Pozzo, Charles L. Raison, Ali John Zarrabi, Barbara O. Rothbaum, Wilbur A. Lam

**Affiliations:** 1 Department of Family and Preventive Medicine, Emory University School of Medicine, Atlanta, GA USA; 2 Department of Spiritual Health, Woodruff Health Sciences Center, Emory University, Atlanta, GA USA; 3 Emory University School of Medicine, Atlanta, GA USA; 4 Department of Psychiatry and Behavioral Sciences, Emory University, Atlanta, GA USA; 5 Wallace H. Coulter Department of Biomedical Engineering, Georgia Institute of Technology and Emory University, Atlanta, GA USA; 6 Department of Biomedical Informatics, Emory University School of Medicine, Atlanta, GA USA; 7 Department of Psychology, University of North Texas, Denton, TX USA; 8 Department of Psychiatry, School of Medicine and Public Health, University of Wisconsin-Madison, Madison, WI 53706 USA

**Keywords:** Speech, Ecological momentary assessment, Experience sampling

## Abstract

This article reports on the validation of Fabla, a researcher-developed and university-hosted smartphone app that facilitates naturalistic and secure collection of participants’ spoken responses to researcher questions. Fabla was developed to meet the need for tools that (a) collect longitudinal qualitative data and (b) capture speech biomarkers from participants’ natural environments. This study put Fabla to its first empirical test using a repeated-measures experimental design in which participants (*n* = 87) completed a 1-week voice daily diary via the Fabla app, and an identical 1-week text-entry daily diary administered via Qualtrics, with diary method order counterbalanced and randomized. A preregistered analysis plan investigated (1) adherence, usability, and acceptability of Fabla, (2) concurrent validity of voice diaries (vs. text-entry diaries) by comparing linguistic features obtained via each diary method, and (3) differences in the strength of the association between linguistic features and their known psychological correlates when assessed by voice versus text-entry diary. Voice diaries yielded more than double the mean daily language volume (word count) compared to text-entry diaries and received high usability and acceptability ratings. Linguistic markers consistently associated with depression in prior research were significantly associated with depression symptoms when assessed via voice but not text-entry diaries, and the difference in correlation magnitude was significant. Word-count-adjusted linguistic patterns were highly correlated between diary methods, with statistically significant mean differences observed for some linguistic dimensions in the presence of these associations. Fabla is a promising tool for collecting high-quality speech data from participants’ naturalistic environments, overcoming multiple limitations of text-entry responding.

## Introduction

The collection of longitudinal self-report data from participants’ natural daily contexts has become a cornerstone of many fields of research, including psychology, medicine, public health, and sociology. This is typically accomplished using methodologies known as daily diary and ecological momentary assessment (EMA; also referred to as experience sampling or ESM). Daily diary and EMA prompt participants to self-report their experiences, behaviors, and other constructs of interest at time intervals determined by researchers, most commonly by sending survey questions to participants’ smartphones. The strengths of these methods are well documented, and include high ecological validity, reduced recall biases, and temporal and contextual sensitivity (Rodríguez-Blanco et al., 2018; Shiffman et al., 2008; Smyth & Heron, 2014; Smyth & Stone, 2003). However, these methods are also accompanied by widely recognized challenges. Foremost among these is participant burden: EMA and daily diary require repeated and naturalistic participant engagement over time, placing a considerably higher burden of time and daily intrusiveness than retrospective questionnaire measures. Correspondingly, these studies are vulnerable to low adherence and can be expensive to administer. A recent meta-analysis of EMA studies found that, while participant incentives (e.g., per-prompt compensation, with a bonus for overall high compliance) substantially increases compliance, published EMA/ESM studies on average report response rates of 79% and dropout rates of 10.5% (Wrzus & Neubauer, 2021).

The presence of qualitative questions in EMA and daily diary protocols exacerbates participant burden. Although qualitative questions (e.g., items that ask participants to describe the details of an emotionally salient event or describe the ways in which a symptom interfered with that day) add valuable thematic and linguistic data points to intensive longitudinal studies, these items take more time and cognitive effort to respond to than scale and multiple-choice question types. Further, the task of typing elaborated responses into a smartphone, or alternatively of getting to a computer to use a keyboard, can pose feasibility and accessibility barriers to providing complete responses. Relatedly, as previously characterized in research on dictation software, many people experience writing and typing as laborious and nonintuitive ways to communicate (Blackley et al., 2020; Smith & Chaparro, 2015; Tsai, 2021). Finally, written responses are inherently subject to self-editing (i.e., participants can revise what they have written), which can constrain validity for some qualitative questions. Many cognitive and emotional processes of clinical interest (e.g., rumination, the repetitive dwelling on a past event; Eisma et al., 2022; Kaplan et al., 2018) are easily “edited out” of typed responses.

The microphones included in all commercially available smartphones pose an intuitive potential solution to these data collection challenges. Voice dictation has become an increasingly common part of peoples’ everyday routines and is now widely used for communication (e.g., sending voice messages to others or making voice memos for oneself) and daily tasks (e.g., using voice assistants such as Siri and Alexa for tasks such as making grocery lists and obtaining directions). Voice dictation may therefore reduce participant burden associated with responding to qualitative EMA and daily diary questions by reducing the time and effort required to provide complete responses and increasing feasibility by allowing participants to respond to researcher questions in the same manner as they would send a voice memo to a friend. Voiced responses also add novel and meaningful data points: in addition to containing the thematic and linguistic information also contained in written responses, voice data include acoustic speech features (e.g., prosody, tone, volume; Ramanarayanan et al., 2022; Robin et al., 2020) that track with a number of psychological symptoms and processes, including personality (Guidi et al., 2019; Polzehl, 2015), depression (Albuquerque et al., 2021; Cummins et al., 2015; Koops et al., 2023), and post-traumatic stress disorder (Broek et al., 2010; Marmar et al., 2019; Vergyri et al., 2015).

## Fabla: An app for collecting spoken responses to EMA and daily diary questions

This article introduces and reports on a validation study of Fabla, a researcher-developed and university-hosted smartphone app that facilitates secure and naturalistic collection of participants’ verbalized replies to researcher questions. In simple terms, Fabla allows participants to respond to researcher-generated questions out loud, as though sending a voice memo to a friend. Fabla was developed via an existing academic–software engineering partnership, the Emory University AppHatchery, and was designed for scientific and clinical research studies that use EMA, daily diary, and naturalistic qualitative or mixed-method study designs.

**Fig. 1:**
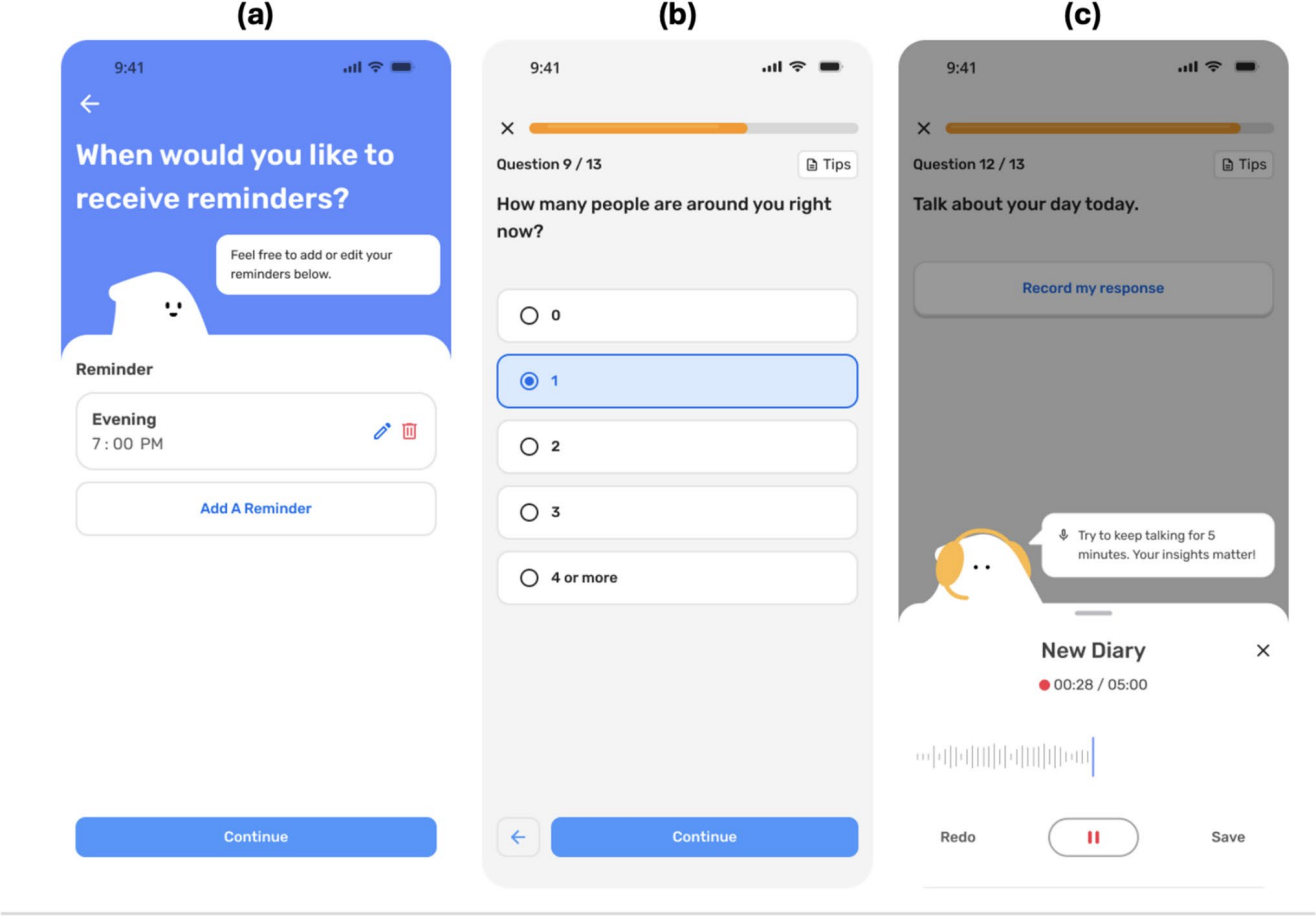
Overview of the Fabla app. *Note*. Figure shows participant view of the Fabla app. Data provided through the app are securely and automatically transmitted through HTTPS requests to Emory University’s Health Insurance Portability and Accountability Act (HIPAA)-eligible Amazon Web Services (AWS) infrastructure. Audio files are saved as.mp3 or.wav formats and automatically titled with a participant identifier and timestamp. Quantitative data can be downloaded as a single CSV file, aggregated by participant identifier and timestamp, for analysis

**Fig. 2:**
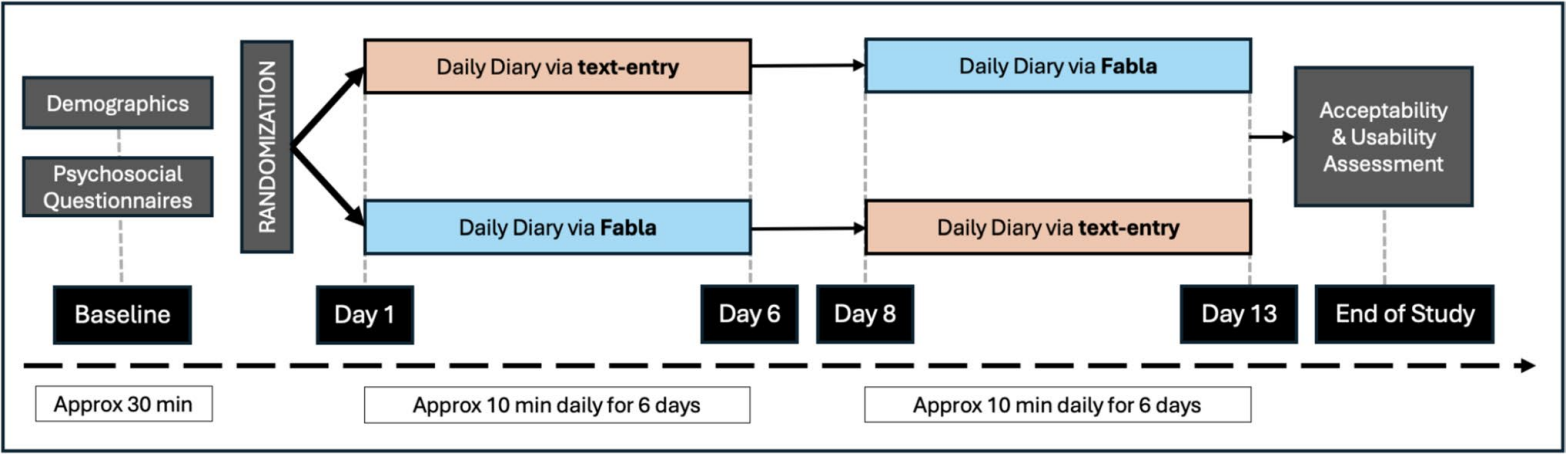
Overview of study procedures

**Fig. 3:**
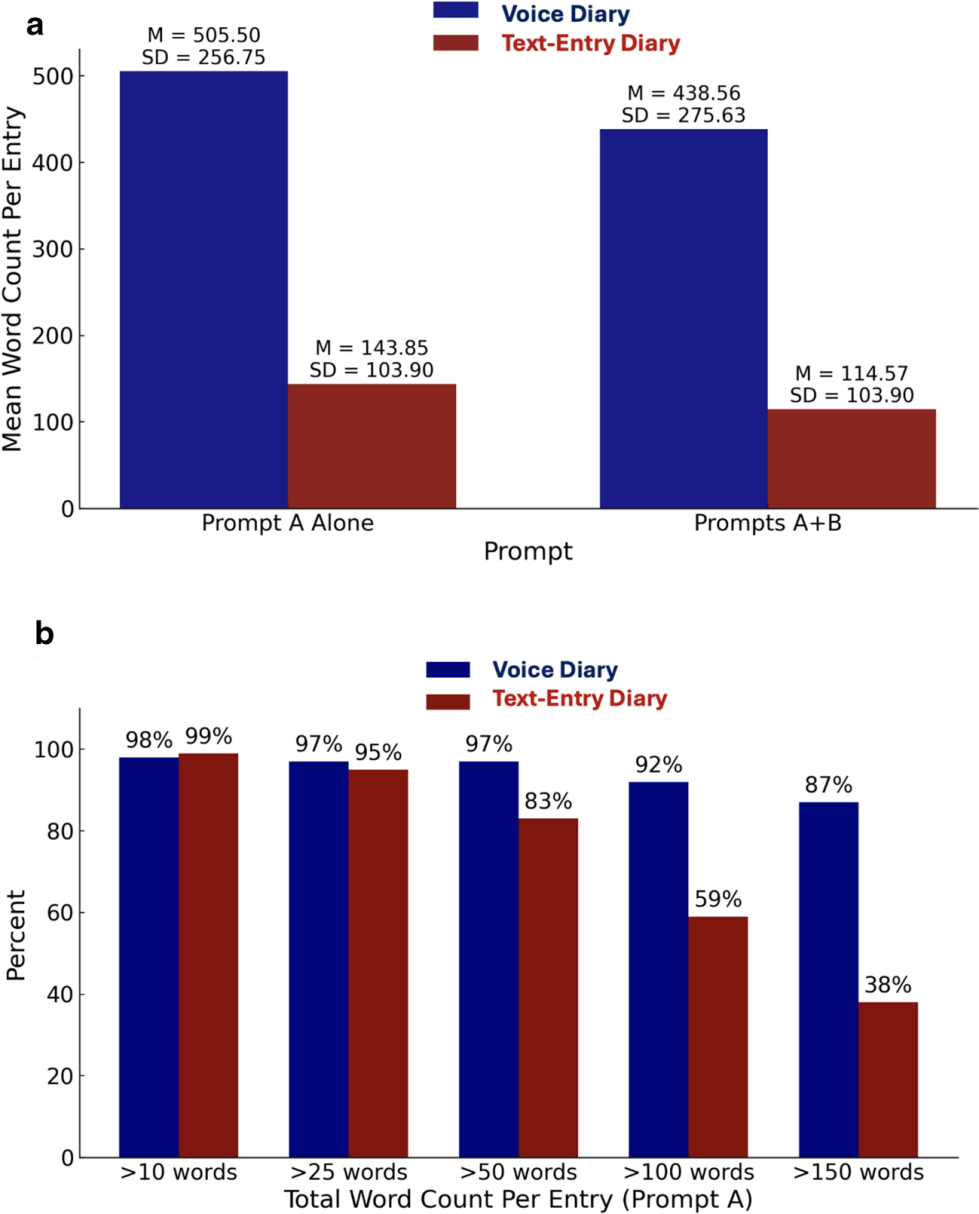
Word count comparison between voice and text-entry diary methods. *Note.* **a** Visualizes average diary word counts for prompt A alone and prompts A+B (with A and B prompts treated as separate entries for descriptive analyses). **b** Visualizes word count differences by cut point for prompt A (the first daily diary question), including all entries with at least one word, *n* = 319 voice diaries and *n* = 308 text-entry diaries

Fabla was developed through formative, user-centered design research conducted with faculty researchers who use ecological assessment or language assessment methodologies (*n* = 15) and non-scientist past participants in EMA or daily diary studies (*n* = 12). These individuals completed needs assessment interviews with the app development team and subsequently provided feedback on prerelease prototypes of Fabla. Guided by these findings, the core functionalities of the app include (1) a customizable reminder notification interface to accommodate any desired assessment schedule (Fig. 1a), (2) an option for multiple-choice and Likert-type items that can accompany voice recording prompts and be customized by researchers (Fig. 1b), and (3) voice diary prompts that can be customized by researchers (Fig. 1c). Additionally, the app’s user interface was designed to balance features that make the app feel “inviting” with avoiding images that may elicit measurement reactivity. Finally, in the context of mounting scientific interest in predictive speech analytics, the data security infrastructure was developed to meet regulatory requirements for personally identifiable information (PII) in the United States, where these authors reside.

### Methodological foundations

Methodologically, the Fabla app is an extension and innovation of EMA and daily diary methodologies, which are already well established (Mehl & Conner, 2011; Shiffman et al., 2008). These established methods primarily collect quantitative and written, open-response data from participants. To our knowledge, only one EMA platform, m-Path, has added the ability for participants to respond via audio recordings (Mestdagh et al., 2023). Fabla was developed with the goal of elevating voice as a central mode of self-report. We sought to create a user platform that would promote natural, conversational responses and an infrastructure built to meet regulatory standards for collecting and transmitting speech data in clinical research in several countries worldwide, including the United States (see *Technical specifications*).

Fabla also takes inspiration from other established methods for the collection of participant speech, such as the stream-of-consciousness (SOC) task (Holleran & Mehl, 2008; Pennebaker & King, 1999) and the Electronically Activated Recorder (EAR) (Kaplan et al., 2020; Mehl, 2017). The SOC is a laboratory task in which participants are instructed to speak their thoughts into a voice recorder as they come to mind, and assumes that when participants follow their spontaneous stream of thought, the content and style of the language produced provides direct insight into their psychological world. The SOC task has been used to investigate linguistic associations with a wide range of psychological attributes and processes, including personality, cognitive schemas, and psychological distress and coping (Bourassa et al., 2019; Lazarević et al., 2020; Raffaelli, 2020; Sripada & Taxali, 2020). The EAR is a smartphone app that passively makes brief and periodic audio recordings from participants’ momentary environments (e.g., a 30-s recording every 12 min over the course of 3 days). Participants are unaware of when the EAR app is or is not recording, and the app captures a sample of participant speech naturalistically, as speech occurs during social interactions with others (Kaplan et al., 2020; Mehl, 2017; Mehl & Holleran, 2007). Finally, Fabla’s development was further inspired by researcher groups who have recently collected speech recordings from participants in their natural environments using other tools, such as handheld digital voice recorders lent to participants (Mammen et al., 2019) or through recorded telephone calls made by participants from their homes (Provost et al., 2024).

### Technical specifications and data security 

Fabla was developed in Flutter for iOS and Android platforms and uses a Health Insurance Portability and Accountability Act (HIPAA)-eligible Amazon Web Services (AWS) infrastructure to transmit and store the data. Researchers can set up a study with Fabla through a web interface connected to the app’s backend system. The study configuration—including the items and response options, ordering and logic, and scheduling protocols—is managed through this web portal. Once configured, when the user enters the study, the app automatically downloads the relevant study instructions and sets up the participant-facing content accordingly. Study protocols containing the number, duration, prompts, and time delivery of the questions are retrieved by participants'personal devices using study and participant identification numbers. Local notifications adjusted to the participants’ time zones are delivered to participants to alert them when responses are available and due. Available question types include quantitative (e.g., single and multiple-choice, scale, rating), text-entry, and audio or video recordings. Each recording session involves participants responding to prespecified prompts, with audio files saved in.mp3 or.wav formats. All data are securely and automatically transmitted through HTTPS requests to Emory University’s AWS account. AWS API Gateway authenticates requests and routes data to DynamoDB and S3. The data are encrypted both in transit and at rest using AES-256 encryption, ensuring secure data handling. Quantitative data can be downloaded as a single CSV file for analysis.

Fabla was developed in the United States for compliance with US laws and regulations for the protection of speech data. To facilitate international research, data protection and privacy procedures were also guided by review of the General Data Protection Regulation (GDPR, European Union), the 2018 UK Data Protection Act (UK GDPR, UK), the UK Privacy and Electronic Communications Regulation, implementing the ePrivacy Directive (PECR, UK), the AU Privacy Act 1988 (Australia), and the NZ Privacy Act 2020 (New Zealand). However, compliance with local relevant laws is typically determined in the context of specific research procedures being used in any given study. As of the date of publication of this article, the Fabla app has been designated as compliant with PII protections in audio file data handling by the Security Review team at Emory University, and is institutional review board (IRB)-approved for use in multiple US-based universities and Veterans Affairs Medical Centers, as well as by an IRB in Australia for a study that will collect data in Australia, New Zealand, and the United Kingdom.

### The present study

The overarching goal of this research was to empirically test the Fabla app by characterizing its acceptability, usability, and adherence, as well as similarities and differences between voice daily diaries collected using the Fabla app and text-entry daily diaries collected via a web-based platform that has been widely used in daily diary and EMA research (Canty et al., 2024; Cui et al., 2022; Neupert & Bellingtier, 2018; Palitsky, 2020). This study used a counterbalanced, repeated-measures experimental design in which participants were asked to complete a 1-week text-entry diary, and a 1-week voice diary using the Fabla app, with all participants completing both diary methods and the order of diary method randomized between conditions.

This study had three preregistered aims. In Research Question 1 (RQ1*)*, we investigated the adherence, usability, and acceptability of Fabla as a tool for qualitative ecological assessment research. Two additional research questions examined the validity of smartphone-captured voice responses for collecting linguistic features that have been of wide interest in the clinical and health sciences. RQ2 investigated the concurrent validity of voice diaries (as compared to text-entry diaries) by comparing linguistic features obtained via each diary method, using the count-based text analysis program Linguistic Inquiry and Word Count (LIWC-2022; Boyd et al., 2022). RQ3 investigated associations between a prespecified set of linguistic features and their known psychological correlates when assessed by a voice diary compared to a text-entry diary. RQ3 was guided by existing meta-analytic evidence (Holtzman, 2017; Koutsoumpis et al., 2022; Tølbøll, 2019) for linguistic markers of psychological symptoms and processes identified in written and spoken language samples alike. The presence and magnitude of these associations in Fabla-assessed data are therefore useful indicators of Fabla’s ability to collect language markers that have established predictive validity.

## Methods

The study took place from November 5 through November 28, 2023. Procedures used in this research were reviewed and approved by the IRB at Emory University. Per recommendations of Benning et al. (2019), we registered our methods and analytic plan, including variable computation and inclusion criteria for planned analyses, after completing data collection and prior to conducting analyses, on the Open Science Framework at https://osf.io/jymtd/.

### Participants

Participants meeting inclusion criteria for this study were over 18 years old and had adequate English fluency to provide informed consent. Participants were undergraduate students (*n* = 87) at a university in the midwestern United States (*n* = 42), a university in the southeastern United States (*n* = 27), and a university in the southwestern United States (*n* = 18) who enrolled in the research for extra credit.

### Procedures

#### Recruitment

 Participants were recruited during undergraduate courses at three geographically distinct universities, taught by instructors known to the research team but not involved in the development of the Fabla app or this study. During a regularly scheduled class, a member of the research team provided a brief (approximately 10-min) overview of this research study. Participants were informed that they would receive extra credit for participating in the research. An alternative assignment of completing a private daily diary not used in the research study was provided for students who desired extra credit but did not wish to participate in this study. Instructors did not have access to diaries provided for the study or the alternative assignment.

#### Data collection procedures

 This study used a repeated-measures experimental design in which a 6-day text-entry diary and a 6-day Fabla voice diary were counterbalanced across two randomly assigned conditions (Fig. 2).

Students were provided a link to an online consent form administered via Qualtrics. Those who consented to participate were then directed to complete a baseline questionnaire including demographics and multiple psychosocial questionnaires (see *Measures*), with several attention check items embedded throughout the survey (e.g., “If you are reading this, please select ‘Slightly agree.’ This is not a trick question.”).

All participants who completed the baseline questionnaire were subsequently randomized using the *=RAND()* function in Excel version 16.89.1, which generates pseudo-random numbers from the uniform distribution. Participants were randomized to one of two counterbalanced conditions, both of which consisted of a 6-day voice daily diary using the Fabla app and a 6-day text-entry daily diary using Qualtrics, a software platform that has been widely used in daily diary and EMA study designs (e.g., Canty et al., 2024; Cui et al., 2022; Palitsky, 2020). The two conditions differed only in the order in which daily diaries using each method were collected. The within-subjects repeated-measures design allowed for evaluation of individual-difference characteristics as assessed by both methods for each participant, and the counterbalanced design aimed to help account for order effects on outcome variables.

Participants in this research were asked to download the Fabla app to their own smartphone, via either the App Store (for iPhone devices) or the Google Play Store (for Android devices). The Qualtrics version of the daily diary was visually optimized for smartphone, although participants had the ability to complete it from any device with an internet browser. Participants received daily email reminders to complete their diary during both study periods. When using Fabla, participants were additionally able to set personalized reminder notifications at the time(s) of their choice via the app. The study took place from November 5 through November 28, 2023. Participants received an email on Sunday, November 5, notifying them of the condition to which they had been assigned and instructions for completing their daily diary that week. The first daily diary period took place from November 6 to November 11 (Monday through Saturday). On Sunday, November 12, participants received instructions to switch diary methods for the second study week, with no diary completed that day. The second daily diary period took place from November 13 to November 18 (Monday through Saturday), and participants received the end-of-study usability and acceptability survey on Monday, November 19. The end-of-study survey remained open for 10 days.

#### Participant incentivization (extra credit criteria)

To receive extra credit, students were required to complete a minimum of seven of the possible 12 daily diary entries, in any combination. This meant, for example, that a student could elect to complete six text-entry diaries and only one voice diary in Fabla (or the other way around) and be eligible for extra credit. Students were informed of the criteria for receiving extra credit in advance of the research and were instructed that completion of an entry did not have any length requirements, meaning that they could report as little or as much as they had to say in each daily diary. This extra credit criterion aimed to incentivize > 50% compliance in the daily diary procedures overall, without incentivizing the use of either method or influencing the quantity or nature of data provided in the diaries.

#### Measures

Psychosocial questionnaires completed by participants at baseline included a detailed demographics questionnaire, the 44-item Big Five Inventory (BFI) (John et al., 2008), and the General Depression scale from the Inventory of Depression and Anxiety Symptoms (IDAS-II; Watson et al., 2012), from which the two items assessing suicidality were omitted, for a total of 18 items.

In the end-of-study survey, *usability* was assessed using the System Usability Scale (SUS; Lewis, 2018) and evaluated based on previously established benchmarks (Lewis & Sauro, 2018). *Acceptability* was assessed quantitatively with two items on a scale of 0–10 (“How convenient was the Fabla app?” and “To what extent would you recommend the Fabla app to others who are participating in a daily diary research study in the future?”), and with a binary item that asked participants to indicate which method of providing a diary they preferred (speaking or typing). *Privacy concerns* about Fabla were assessed with two questions ranked by participants on a scale of 0–10: “When using Fabla, how concerned were you about being overheard by others?” and “When using Fabla, how difficult was it for you to get to a private enough location that you felt comfortable speaking freely?” The survey concluded with open-ended, qualitative items asking participants to share likes and dislikes about each diary method.

The daily diary consisted of 10 single-item Likert-type questions (not analyzed in the present study) followed by two open-ended qualitative diary items captured via text-entry in Qualtrics or voice recording in Fabla. Participants were also asked to indicate the number of people around them at the time of the entry and to check the location option that best described where they were when completing the entry (at home or in my dorm room; in someone else’s home or dorm room; outside; in a car, bus, or other transportation; in a public place). Qualitative items were identical for both diary methods and consisted of two open-ended prompts. The first prompt, referred to as “prompt A,” was provided each day and asked participants to talk (or write) about their day. The second prompt, “prompt B,” probed for more specific content: on alternating days, prompt B queried daily social connectedness (B1) and daily stressors (B2). Diary prompts are provided in Table 1. Items were uniquely developed for the present study, although the instructions to participants were informed by those used in prior research on longitudinal stream-of-consciousness laboratory speaking tasks and daily expressive writing tasks (Lazarević et al., 2020; Pennebaker & Smyth, 2016; Sripada & Taxali, 2020). In the voice diary condition, participants could see a timer displaying the amount of time elapsed while recording. This was not available in the text-entry diary condition; participants needed to look at the clocks on their computer or phone for information about time elapsed while typing.

**Table 1:** Daily diary prompts

Prompt	Voice diary (Fabla app)	Text-entry diary (Qualtrics)
**A** (daily)	**Talk about your day today.** Please try to talk without stopping for about 5 minutes. Talk about whatever comes to your mind, as if you were sharing with a friend. Do not worry about pauses, stutters, or having the right things to say. We are interested in anything about your experience today that you are willing to share.	**Write about your day today.** Please try to write without stopping for about 5 minutes. Write whatever comes to your mind, as if you were sharing with a friend. Do not worry about spelling, grammar, or having the right things to say. We are interested in anything about your experience today that you are willing to share.
**B1** (alternating days)	**Talk about a time today when you felt understood or cared for by others, no matter how small.** Please try to talk without stopping for about 5 minutes. Talk about whatever comes to your mind, as if you were sharing with a friend. Do not worry about pauses, stutters, or having the right things to say. We are interested in anything about your experience today that you are willing to share. (It is OK if there is overlap with what you talked about for the previous question.)	**Write about a time today when you felt understood or cared for by others, no matter how small.** Please try to write without stopping for about 5 minutes. Write whatever comes to your mind, as if you were sharing with a friend. Do not worry about spelling, grammar, or having the right things to say. We are interested in anything about your experience today that you are willing to share. (It is OK if there is overlap with what you wrote for the previous question.)
**B2** (alternating days)	**Talk about today’s most stressful experience, no matter how small.** Please try to talk without stopping for about 5 minutes. Talk about whatever comes to your mind, as if you were sharing with a friend. Do not worry about pauses, stutters, or having the right things to say. We are interested in anything about your experience today that you are willing to share. (It is OK if there is overlap with what you talked about for the previous question.)	**Write about today’s most stressful experience, no matter how small.** Please try to write without stopping for about 5 minutes. Write whatever comes to your mind, as if you were sharing with a friend. Do not worry about spelling, grammar, or having the right things to say. We are interested in anything about your experience today that you are willing to share. (It is OK if there is overlap with what you wrote for the previous question.)

### Data analytic strategy and computation of variables

This data analysis plan was preregistered on the Open Science Framework at https://osf.io/jymtd/. As noted in the preregistration, the average System Usability Scale (SUS) scores and app acceptability ratings (included in RQ1) had been descriptively examined by the app engineering team for formative evaluation purposes prior to the creation of the preregistered analytic plan. All other analyses were preregistered after data collection ended but prior to completing any analyses. Analyses were conducted using IBM SPSS version 29.

- ***RQ1. What is the adherence, usability, and acceptability of a smartphone-based daily voice diary?***

All participants who passed attention check items in the baseline survey and completed at least one diary entry (using either method) were included in adherence analyses. Adherence was operationalized in the following ways, acknowledging that different types and thresholds of adherence may be distinctly applicable for different applications: (1) total number of entries completed using each method, (2) completion of more entries using one method versus the other, (3) completion of > 50% of entries (the threshold incentivized in this study), and (4) meeting thresholds of > 25, > 50, > 100, and > 150 words in entries. Correspondingly, (1) the total number of entries from text-entry versus voice diaries was compared using a two-way (method × order) analysis of variance (ANOVA); (2) the total number of individuals who completed more text-entry than voice diaries was compared using a chi-square (method × order) test; (3) the number of participants with > 50% adherence to text-entry versus voice diaries was compared using a chi-square (method × order) test; and (4) the frequencies of entries with > 10 words, > 25 words, > 50 words, > 100 words, and > 150 words were examined descriptively.

All participants who completed the end-of-study survey and passed attention check items in the survey were included in usability and acceptability analyses. Usability was examined by computing and descriptively examining total SUS scores for Fabla. Acceptability was examined via descriptive statistics of acceptability ratings and items assessing privacy concerns (provided in *Measures*). Binary preferences for text-entry diary versus voice diary were evaluated with a chi-square test of independence. Open-ended feedback provided via the end-of-study survey was reviewed to contextualize observed quantitative results. Contexts of use were characterized descriptively by aggregating the frequencies with which participants completed text-entry and voice diaries in each possible location, and the number of individuals nearby reported by participants at the time of each diary entry.

- ***RQ2. How do linguistic features obtained using a voice diary compare to linguistic features obtained using a text-entry diary?***

*Linguistic data preparation.* Linguistic features were computed using Linguistic Inquiry and Word Count (LIWC-2022) (Boyd et al., 2022). Text-entry diary entries were directly exported for LIWC analyses; voice diary entries were initially transcribed using the automated transcription service Otter.AI, with each transcript subsequently verified by a trained research assistant, who listened to each raw audio file and corrected transcripts as necessary to ensure verbatim accuracy. Although this study did not systematically document the nature and frequency of needed corrections, we direct interested readers to several recent investigations of the accuracy of Otter.AI and other automated transcription programs, including OpenAI Whisper and AWS Transcribe (Graham & Roll, 2024; Naffah et al., 2025; Seyedi et al., 2023). Twenty-six of the 117 total LIWC-22 variables were a priori selected for computation and analyses based on their frequent use as indicators of psychological processes in previous studies (Chung & Pennebaker, 2018; Koutsoumpis et al., 2022; Tølbøll, 2019), summarized in Table 2.

**Table 2:** Selected LIWC-2022 variables

LIWC-2022 variable	Definition and examples
*Summary variables*	
Total word count	Total linguistic volume
Big words	Percentage of words using seven letters or longer
Dictionary words	Percentage of words found in the LIWC dictionary
*Linguistic dimensions*	
I-talk	First-person singular pronouns; I, me, my myself
We-talk	First-person plural pronouns; we, ours, us, lets
She/they	Third-person singular pronouns; he, she, her, his
They/them	Third-person plural pronouns; they, their, them
Verbs	Is, was, be, have
Adjectives	More, very, other, new
Negations	Not, no, never, nothing
*Psychological processes*	
Cognition	Is, was, but, are
Affect	Good, well, new, love
Positive tone	Good, well new, love
Negative tone	Bad, wrong, too much, hate
Emotion	Good, love, happy, hope
Positive emotion	Good, love, happy, hope
Negative emotion	Bad, hate, hurt, tired
Swear words	Shit, fuckin*, fuck, damn
Social behavior	Said, love, say, care
Social referents	You, we, he, she
Health	Medic*, patients, physician*, health
Perception	In, out, up, there
Time orientation	
Past focus	Was, had, were, been
Present focus	Is, are, I’m, can
Future focus	Will, going to, have to, may

Definitions and examples are provided as published in the LIWC-2022 Manual (Boyd et al., 2022). Indentation is used to indicate subcategories; for example, the category “emotion” comprises all words in the language dictionaries for both “positive emotion” and “negative emotion.” Word count was computed as the total number of distinct words in the text-entry sample, based on separation by spaces in a transcript. All other variables are computed by LIWC as the proportion of words from that language category out of the total word count (e.g., if a person provided 10 negative emotion words out of 100 total words, the LIWC output for negative emotion words would be.10 or 10%). An asterisk (*) denotes the acceptance of all other letters or hyphens following its appearance

These LIWC-2022 variables were separately computed for voice diary and text-entry diary responses and, for RQ2 analyses, aggregated within participant by averaging. For each diary method, LIWC-2022 variables were computed for the following: (1) responses obtained for only prompt A, the first and most general daily diary prompt, which recurred daily, and (2) responses obtained for prompts A and B (including B1 and B2 on alternating days), combined. Given that the B1 and B2 prompts seemed likely to elicit elevations on language categories of interest due to the nature of the prompts themselves (e.g., being asked about stress might elicit negative emotion words; being asked about a social experience might elicit social words), overall language features were not computed separately for prompts B1 and B2 for this study. In summary, for each participant, four LIWC-2022 language summaries were computed: (1) voice diary condition prompt A alone, (2) voice diary condition prompts A+B1+B2, (3) text-entry diary condition prompt A alone, and (4) text-entry diary condition prompts A+B1+B2.

#### Analyses

Consistent with the preregistration, RQ2 analyses included participants who passed attention checks at baseline and provided at least one text-entry diary and one voice diary. Linguistic differences between text entries and voice entries (methods) were examined using repeated-measures ANOVA models with method (text-entry vs. voice), condition (order of methods), and prompt type, another potential source of method variance (A alone vs. A+B1+B2), included as independent variables, and LIWC-derived language variables as dependent variables, with complete case analysis (i.e., listwise deletion) used for missing data given the novelty of the present analyses and importance of complete data. Order effects were examined, followed by an analysis of differences in linguistic features based on method, prompt, and the interaction of method and prompt, adjusting for order effects. Pearson correlations were additionally computed for the voice-assessed and text-entry-assessed version of each language variable. These correlations are reported to contextualize systematic differences in language in the context of significant associations, although they were not included in our preregistered analysis plan.

Our preregistration additionally proposed subsequent exploratory analyses examining the potential impact of additional psychological and demographic variables by adding each of them to the tested model as covariates in separate analyses. However, these exploratory analyses were found to have low statistical power, and following the recommendations of Alderson (2004), we have opted to eschew reporting them and instead suggest this as an important direction for research in future studies that are adequately powered to detect these effects.

- ***RQ3: How does the strength of the association between linguistic features and their psychological correlates differ when language is assessed through voice diaries compared to text-entry diaries?***

Preregistered analyses examined one clinical psychological dimension (depression) and one nonclinical psychological dimension (five-factor personality) that have demonstrated meta-analytic evidence of correlation with linguistic variables, summarized in Table 3.

**Table 3:** Selected psychological constructs and linguistic correlates

Variable	Linguistic markers
Depression	Positively associated with first-person pronouns^a^ and negative affect language^b^
Extraversion	Positively associated with positive emotion words and social behavior words^c^
Openness	Positively associated with longer words^c^
Conscientiousness	Positively associated with achievement words and negatively associated with negative emotion words^c^
Agreeableness	Negatively associated with negative emotion words and swear words; positively associated with social referents and positive emotion words^c^
Neuroticism	Positively associated with negative emotion words and first-person pronouns^c^

^a^ Meta-analytic evidence summarized in Holtzman (2017) . ^b^ Meta-analytic evidence summarized in Tølbøll, 2019, and review-level evidence for speech data specifically summarized in Koops et al. (2023) . ^c^ Meta-analytic evidence summarized in Koutsoumpis et al. (2022)

BFI personality domain scores and an IDAS-II mean General Depression score (assessed at baseline the day before beginning the daily diary, see *Measures*) were computed for each participant. Bivariate Pearson correlations and confidence intervals were computed between these six psychological variables and prespecified linguistic variables (Table 3) from each diary method using prompt A only, given that prompt B may introduce specific language effects as a function of prompt content (i.e., asking about social activity or stress). Differences in significance of association between psychological variables and their linguistic correlates were examined descriptively, as well as using Fisher’s *r-*to-*z* transformation and Steiger’s (1980) equations 3 and 10 to compute the asymptotic covariance of the estimates for an asymptotic *z* test (Lee & Preacher, 2013) to examine significant differences in correlation (e.g., whether the associations between depression and negative emotion language are significantly different when using different diary methods).

#### Multi-trait multi-method (MTMM) validity

 The analytic approach used in RQ2 and RQ3 aim to provide an initial assessment of the MTMM validity (Campbell & Fiske, 1959) of naturalistically completed voice diaries. Differences and similarities in language values are examined in relation to (1) methods, using a repeated-measures approach that examined the effects of voice versus text-entry as well as different prompts, and enabled the parsing of effects due to stability of language use across any given individual (RQ2). Complementing this analysis, a Pearson correlation examined the extent to which language use with one method related to language use with the other, identifying overall similarities even in the presence of potential systematic differences. With regard to the (2) “multi-trait” component of MTMM, associations between language values produced by different methods across different individual-difference variables (i.e., individual-level “traits” of depression and Big Five personality domains) were examined by means of Pearson correlations among language outcomes aggregated within individuals (RQ3). Although the traits tested in the present study represent only some of the numerous possible individual-level traits that may impact language use, the resulting multi-trait-multi-method matrix is provided in Fig. 6, with additional reporting provided in Supplementary Document Table S5.

#### Corrections for multiple comparisons 

Although not included in our preregistered analysis plan, given the high number of tests reported in this study, we conducted a post hoc Benjamini–Hochberg correction (Benjamini & Hochberg, 1995) using an acceptable false discovery rate of 0.05 for the following groups of tests: main effects of diary method on language outcomes (RQ2), correlations between voice-assessed and text-assessed language variables (RQ2), and correlations between self-report inventories and language variables (RQ3). The *p* values within each of these three sets of tests were ranked from lowest to highest, and the correction was applied by dividing the rank of the *p* value by the total number of tests, and then multiplying the quotient by the predetermined false discovery rate of 0.05. This number was treated as the significance threshold for each respective test. The lowest-ranking number that met the significance quotient was used as a cutoff, such that every test whose significance value ranked lower (i.e., higher *p* value) was not regarded as significant.

## Results

The data and syntax needed to reproduce reported analyses are provided on the Open Science Framework (OSF) at https://osf.io/q258p/. Supplementary Document Tables S1–S7 are available on this OSF page and are also appended to this manuscript.

### Sample characteristics and participant attrition

Sample characteristics are provided in Table 4. Twenty participants who completed the baseline survey withdrew from the study prior to receiving the end-of-study survey due to “no longer needing extra credit” and thus did not complete the usability and acceptability assessment, resulting in completed acceptability and usability assessments for a subset of 67 individuals. Participants providing partial data (e.g., baseline data and diary entries) up to the point of the end-of-study survey were included in analyses according to the inclusion criteria specified in the preregistered data analytic plan. Examination of the demographic characteristics of non-completers found that they did not differ from those who completed the study.

**Table 4:** Sample characteristics

Variable	Value
Age (years), *M* (*SD,* range)	20.52 (1.11, 18-24)
Gender, *n* (%)	
Woman	66 (75.9%)
Man	21 (24.1%)
Other	0 (0%)
Race/ethnicity, *n* (%)	
White	52 (60%)
Asian or Asian American	17 (20%)
Black or African American	7 (8%)
Hispanic or Latinx	6 (7%)
Middle Eastern or North African	3 (3%)
Other	2 (2%)
Persons with a disability, *n* (%)	11 (13%)
Living situation, *n* (%)	
Off-campus housing	67 (77%)
Fraternity or sorority housing	10 (11%)
Campus residence hall	10 (11)
“I have a bedroom all to myself”	75 (86%)
“I share a bedroom with one or more others”	12 (14%)
IDAS-II General Depression^+^, *M*, (*SD,* range)	50.50 (15.81, 22.22–93.33)

Sample characteristics are reported for participants who passed attention checks and provided at least one diary entry (n = 87). ^+^For descriptive purposes, given omission of two suicidality items from the 20-item IDAS-II General Depression (GD) scale, a prorated GD score was computed for each participant as [Partial_Raw_Score*20]/18. Using these prorated scores and the balanced screening cutoff (Stasik-O’Brien et al., 2019), 36.8% of the sample (32 participants) would screen positive for MDD, while 52.9% (46 participants) would screen positive for any internalizing disorder. Most participants who would screen positive for MDD had scores in the “mild” symptom range, with only 12.6% of participants with scores in either the “moderate” or “severe” range.

- ***RQ1. What is the adherence, usability, and acceptability of a smartphone-based daily voice diary?***

#### Adherence: number of entries

Participants (*n* = 87) provided comparable numbers of voice and text-entry diaries overall. Voice diaries yielded *M = *3.59 (*SD* = 1.91) entries per participant, and text-entry diaries yielded *M = *3.23 (*SD = *2.04) entries per participant. A two-way (method × order condition) ANOVA evaluated whether the method order (text-entry first or voice first) affected the total number of entries provided. Participants provided a greater number of entries for the method that they received first: the average number of text-entries was approximately one entry higher when text-entry was presented first (*M*_text-entry_ = 3.93, *SD*_text-entry_ = 1.80 vs. *M*_voice_ = 2.58, *SD*_voice_ = 2.06), while the opposite was true when the voice diary was presented first (*M*_voice_ = 4.13, *SD*_voice_ = 1.47 vs. *M*_text-entry= _3.00, *SD*_text-entry_ = 2.16). There was a significant interaction between method and condition, *F*(1,85) = 30.81, *p* <.001, η_p_^*2*^ =.27, such that when text-entry was presented first there was greater drop-off as participants transitioned to the voice diary, but when voice diary was presented first there was a smaller decrease in entries when participants switched to text-entry. No significant main effect of method was observed on total number of diary entries, *F*(1,85) = 1.96, *p* =.165, η_p_^2^ =.02. Consistent with these results, a chi-square test revealed non-independence between which method yielded the most diaries and which method was presented first. Participants who received voice diaries first were more likely to complete more voice diary entries overall, while those who received text-entry diaries first were more likely to complete more text-entry diary entries overall, χ^2^(2, *N* = 87) = 22.82, *p* <.001. Finally, a chi-square test examining whether condition assignment was independent from attainment of > 50% compliance (the number required to receive extra credit) was not statistically significant, χ^2^(1, *N = *87) = 0.03, *p = *.869, suggesting that condition assignment did not affect the likelihood of attaining incentivized completion.

#### Adherence: language volume 

Voice diaries yielded more than the twice the language volume (word count) of text-entry diaries, as shown in Fig. 3a. Correspondingly, while a comparable proportion of voice and text-entry diaries exceeded the >10, >25, and > 50 language production cut points, much higher proportions of voice entries satisfied the >100 and >150 language production cut points (Fig. 3b).

#### Usability and acceptability

Valid usability and acceptability data were available for 65 participants who passed all attention checks. Participants provided an average usability rating of *M = *81.12 (*SD = *11.21) for the Fabla app, indicating very high usability in the 90th to 95th percentile according to scale benchmarks (Lewis & Sauro, 2018).

On a scale of 0–10, participants on average gave the Fabla app a convenience rating of *M = *7.98 (*SD = *1.85) and a recommendation to others participating in daily diary research studies of *M = *7.87 (*SD = *1.96). When asked to make a binary choice of which method they preferred for keeping a daily diary (voice vs. text-entry), about three quarters of participants (72.3%) reported that they preferred to speak their diaries, which was statistically significant, χ^2^(1, *N* = 63) = 15.25, *p* <.001. When asked to rate their level of concern for being overheard while using the app (0 = not at all concerned, 10 = very concerned), participant ratings of *M = *4.08 (*SD = *2.98) indicated moderate awareness of this concern. When asked how difficult it was to get to a private enough location that they felt comfortable speaking freely while using the app (0 = not at all difficult, 10 = very difficult), participant ratings of *M = *3.00 (*SD = *2.69) indicated that most participants experienced low levels of difficulty in attaining comfortable levels of privacy.

Open-ended qualitative feedback provided by participants further contextualize these results. When asked what they liked about the voice diary method and the Fabla app, several participants discussed the “natural” feeling of verbalizing their thoughts. As one participant put it, “I loved how easy it was to keep up with my thoughts when talking compared to when typing.” In the words of another participant, “I liked that for the voice diary I just recorded and was able to just talk. Felt more natural and I definitely shared more.” Other participants also highlighted the efficiency of talking compared with writing, noting “It was just easier” and “I liked how I could just talk in one constant stream of thought that was being recorded…Typing out events took a bit longer in my opinion.” When asked what they disliked about voice-recording diaries, a few participants expressed privacy concerns (“Sometimes, I was in a location that made it difficult to speak aloud”; “I preferred to write it because I live with a lot of roommates and it didn’t feel as confidential when I was recording the audio diaries.”). Other participants reflected on the process differences between speaking and writing. In one participants’ words, “Typing it out made me reflect on what I was trying to say more seriously…I thought less critically [with the voice diary].” Another participant expressed a preference for writing, stating “I liked how my text was more professional, especially if someone will be reviewing my report on my day.”

#### Use contexts 

The social contexts in which participants completed their diaries were similar across the two diary methods. Overwhelmingly, participants completed their entries at home (86.2% of voice diaries and 77.4% of text-entry diaries), with smaller proportions completed in someone else’s home (6.7% of voice diaries and 6.5% of text-entry diaries), outside (2% for both methods), and in a car or other form of transportation (2.5% of voice diaries and 1.7% of text-entry diaries). The largest differences observed were for diaries completed in a public place: only 2.5% of voice diaries were reported by participants as completed in public, compared to 10.4% of text-entry diaries. Participants also reported slightly higher rates of completing text diaries with one or more other people around (29.7%) compared to voice diaries (24.2%). There were no instances of voice diaries that could not be transcribed due to background noise.

- ***RQ2. How do linguistic features obtained using a voice diary compare to linguistic features obtained using a text-entry diary?***

A repeated-measures ANOVA revealed significant multivariate within-subject effects of diary method, *F*(26,32) = 28.290, *p* < 0.001, and prompt type, *F*(26,32) = 45.247, *p* < 0.001. Additionally, there was a significant interaction between diary method and prompt type, *F*(26,32) = 6.849, *p* < 0.001. However, there was no significant effect of method order *F*(26,32) = 1.216, *p* = 0.297. For descriptive purposes, pairwise differences in linguistic variables based on method order are presented in Supplementary Document Table S1 (https://osf.io/jbwu4). Significant main effects of prompt were observed on multiple outcome variables, reported in Supplementary Document Table S2 (https://osf.io/jbwu4), although the direction was not consistent. This suggests that, as anticipated, the nature of the prompt used influenced the language elicited.

### Differences between voice and text-entry diaries

**Fig. 4:**
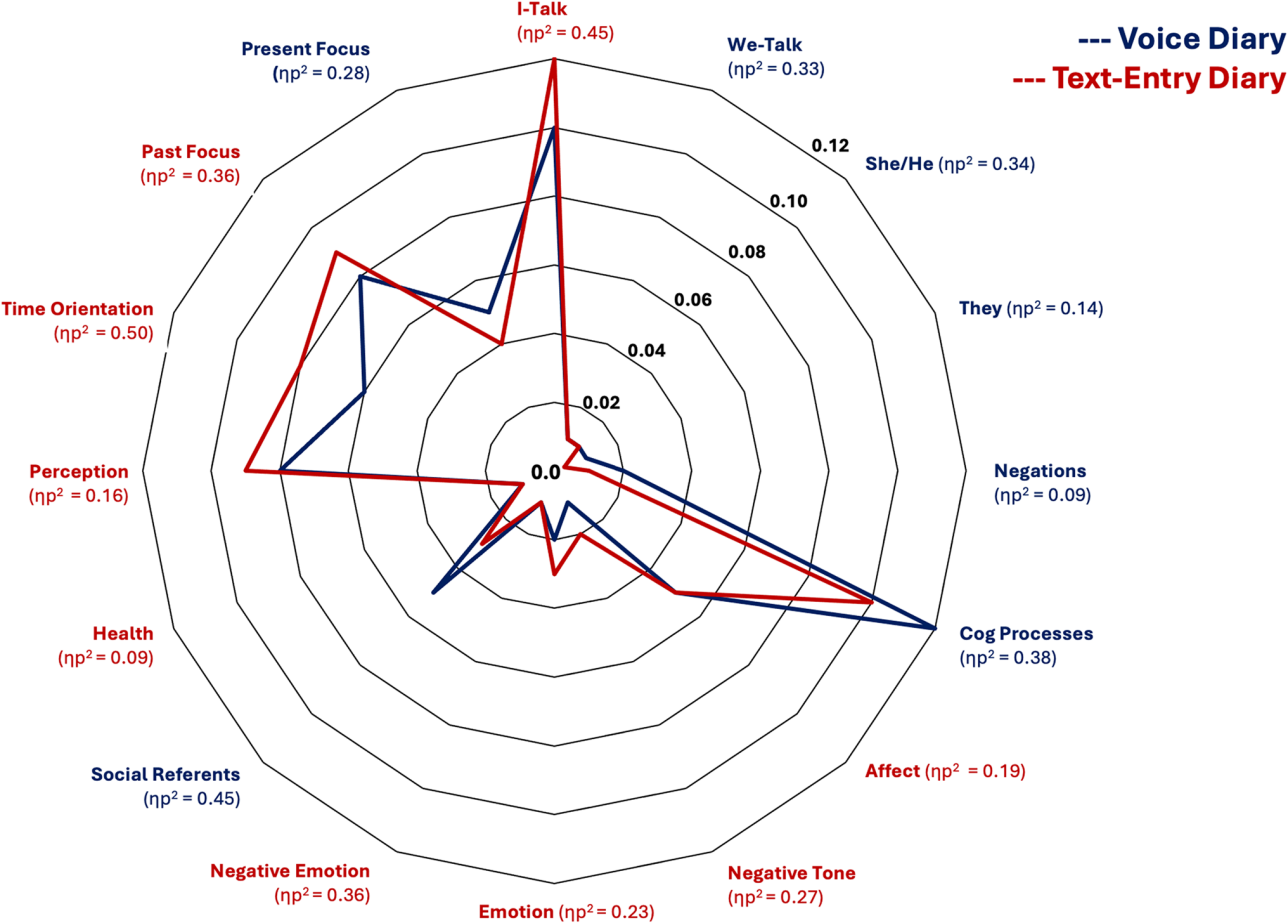
Significant main effects of diary method on language outcomes. *Note.* Radar plot visualizes differences in rates of word-count-adjusted linguistic variables between voice and text-entry diaries. For readability, this figure only includes differences that reached statistical significance using a threshold of *p* <.05 (see Table 5 for full reporting of results, including language variables with nonsignificant differences). Average linguistic category use rates, computed as the proportion of total word count by method, ranged from less than 0.01 (less than 1%) to 0.12 (12%). Variable names colored in red had higher use rates in text-entry diaries; variable names colored in blue had higher use rates in voice diaries. Effect sizes for differences are reported as η_p_^2^

**Fig. 5:**
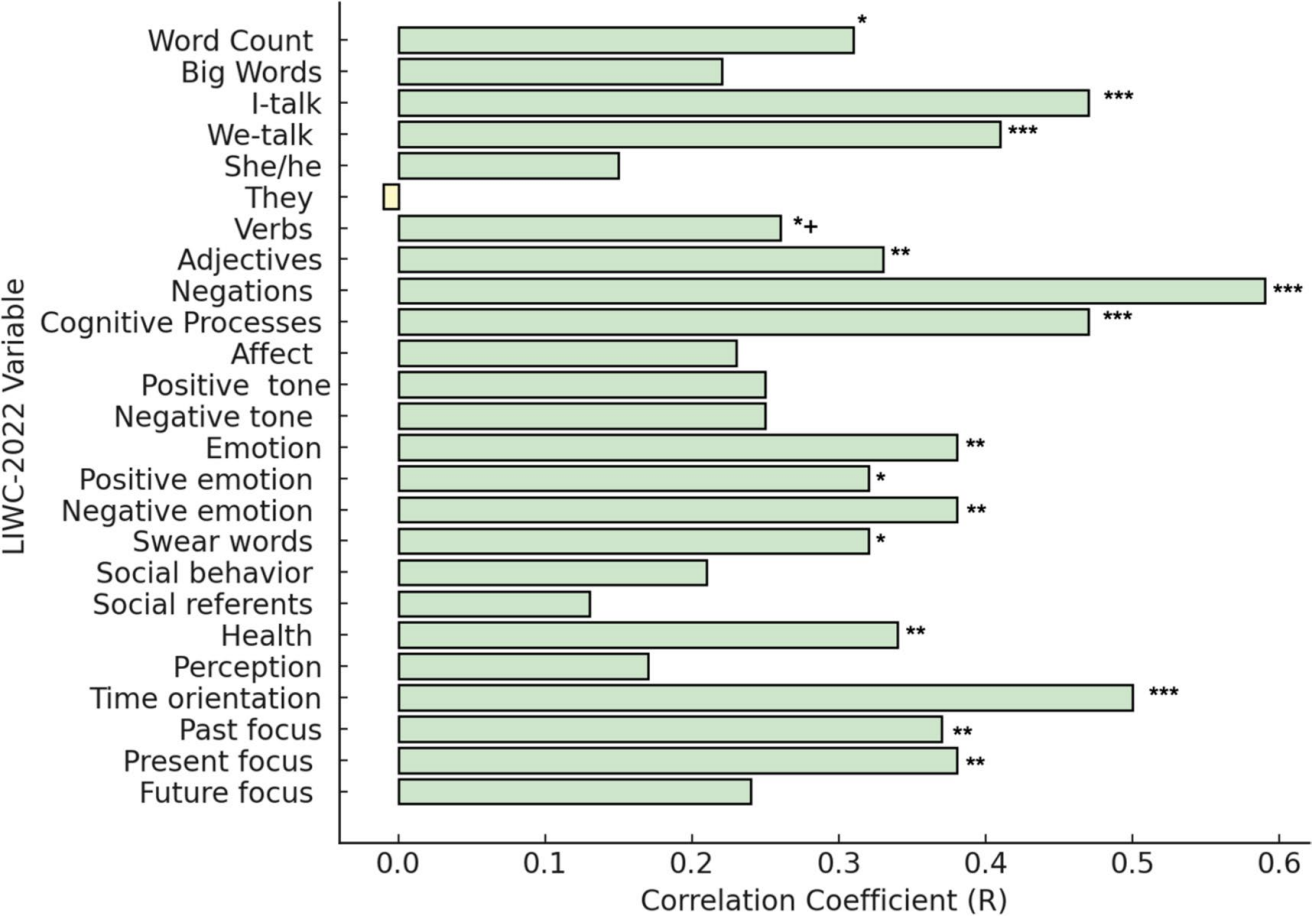
Pearson correlations between voice-assessed and text-entry-assessed language variables. *Note.* Pearson correlations of prompt A language variables assessed via voice and text-entry. *indicates Pearson correlation with value *p* <.05, ** indicates Pearson correlation with value* p* <.01, *** indicates Pearson correlation with value* p* <.001. Following a Benjamini–Hochberg correction accounting for 25 comparisons and resulting in a corrected significance threshold of *p* < 0.03, verbs no longer reached the threshold of significance, as indicated with ^*+^. All daily diary entries with at least one word are included in analyses, reflecting *n* = 319 voice diaries and *n* = 308 text-entry diaries. Means and standard deviations for these correlations are provided in Supplementary Document Table S6. Means, standard deviations, and correlations for prompts A+B are provided in Supplementary Document Table S7 (https://osf.io/jbwu4)

Both voice and text-entry diaries yielded responses with 96% of words found in the LIWC-2022 dictionary, with no significant differences between diary method. Main effects of diary method are reported in Table 5, and significant differences are illustrated in a radar plot (Fig. 4). Comparisons of responses to prompt A versus A+B by method are provided in Supplementary Document Table S3 (https://osf.io/jbwu4). As reported below, differences emerged between voice and text-entry methods for several summary, linguistic, and psychological process variables. However, these differences were observed in the context of significant correlations between voice and text-entry language for each outcome variable (visualized in Fig. 5 and reported with means and standard deviations in Supplementary Document Tables S6 and S7, https://osf.io/jbwu4).

**Table 5:** Main effects of diary method (all outcomes)

	Voice *M* (*SE*)	Text-entry *M* (*SE*)	*F*	*p*	η_p_^2^	Observed power
Word count	469.05 (27.24)	142.05 (9.80)	202.123	< 0.001	0.780	> 0.99
Big words	10.9% (0.002)	12.4% (0.003)	25.126	< −0.001	0.306	0.998
I-talk	10.00% (0.002)	11.60% (0.003)	46.231	< 0.001	0.448	> 0.99
We-talk	1.10% (0.001)	0.60% (0.001)	28.191	< 0.001	0.331	> 0.99
She/he	1.00% (0.001)	0.50% (0.001)	29.865	< 0.001	0.344	> 0.99
They	0.50% (> 0.001)	0.30% (> 0.001)	9.222	0.004	0.139	0.847
Verbs	19.70% (0.002)	19.60% (0.002)	0.162	0.689	0.003	0.068
Adjectives	5.60% (0.002)	5.90% (0.002)	3.396	0.071	0.056	0.441
Negations	1.60% (0.001)	1.40% (0.001)	5.575	0.022	0.089	0.641
Cognitive processes	12.40% (0.003)	10.10% (0.004)	34.313	< 0.001	0.376	> 0.99
Affect	4.50 (0.001)	5.20% (0.002)	13.068	< 0.001	0.187	0.944
Positive tone	3.20% (0.001)	3.30% (0.002)	0.938	0.337	0.016	0.159
Negative tone	1.20% (0.001)	1.80% (0.001)	21.048	< 0.001	0.270	0.995
Emotion	2.10% (0.001)	2.80% (0.002)	16.840	< 0.001	0.228	0.981
Positive emotion	1.20% (0.001)	1.30% (0.001)	1.105	0.298	0.019	0.178
Negative emotion	0.80% (0.001)	1.40% (0.001)	31.339	< 0.001	0.355	> 0.99
Swear words	0.10% (> 0.001)	0.00% (> 0.001)	1.354	0.249	0.023	0.208
Social behavior	2.30% (0.001)	2.10% (0.001)	2.424	0.125	0.041	0.334
Social referents	4.50% (0.002)	3.00% (0.001)	47.212	< 0.001	0.453	> 0.99
Health	0.60% (0.001)	0.90% (0.001)	5.823	0.019	0.093	0.660
Perception	8.20% (0.002)	9.10% (0.003)	10.662	0.002	0.158	0.894
Time orientation	5.80% (0.001)	7.70% (0.003)	57.289	< 0.001	0.501	> 0.99
Past focus	7.80% (0.003)	9.40% (0.003)	32.433	< 0.001	0.363	> 0.99
Present focus	4.50% (0.002)	3.50% (0.002)	21.920	< 0.001	0.278	0.996
Future focus	1.50% (0.001)	1.50% (0.001)	0.029	0.866	0.001	0.053

*n* = *59*. Word count was computed as the total number of distinct words in the text-entry sample, based on separation by spaces in a transcript. All other variables were computed by LIWC as the proportion of words from that language category out of the total word count (e.g., 10 negative emotion words out of 100 total words would be.10 or 10%). All variable means are reported to the nearest 0.01%. All main effects statistically significant at *p < *0.05 remained statistically significant, with a Benjamini–Hochberg correction accounting for 25 comparisons applied

#### Summary variables 

Voice diaries demonstrated a significantly greater word count, averaging over 300 more words per response than text-entry diaries. This finding is consistent with the descriptive analysis of word count differences reported in RQ1, with slight variations in mean numbers attributable to differences in the a priori inclusion criteria set for these analyses. Text-entry diaries contained higher rates of words > 6 letters (“big words”) than voice diaries (see Table 5).

#### Linguistic dimensions

 Compared to text-entry diaries, voice diaries had lower word-count-adjusted rates of first-person singular pronouns (I-talk) and higher word-count-adjusted rates of first-person plural pronouns (we-talk) and negations, although these systematic mean differences occurred in the presence of strong correlations. No differences were observed in other common parts of speech (verbs or adjectives).

#### Psychological processes

 Compared to text-entry diaries, voice diaries had higher word-count-adjusted rates of language involving cognitive processes, present-focus, and social referents, but lower word-count-adjusted rates of language involving past-focus, emotion, negative emotional tone, perception language, and health language. Of these variables, cognitive processes, present focus, past focus, emotion, and negative emotion were significantly associated between methods.

#### Interactions of method and prompt 

Method-by-prompt interactions were observed for several variables, suggesting that differences in linguistic variables elicited by different methods are not independent of prompt characteristics. These variables include word count, big (> 6 letters) words, third-person singular pronouns, adjectives, negations, positive tone, negative tone, emotion words, negative emotion words, social behavior, social referents, and future focus. Values for these interaction effects are presented in Supplementary Document Table S4; simple effects of differences by method within each prompt are presented in Supplementary Document Table S3 (https://osf.io/jbwu4).

- ***RQ3: How does the strength of the association between linguistic features and their psychological correlates differ when language is assessed through voice diaries compared to text-entry diaries?***

**Fig. 6:**
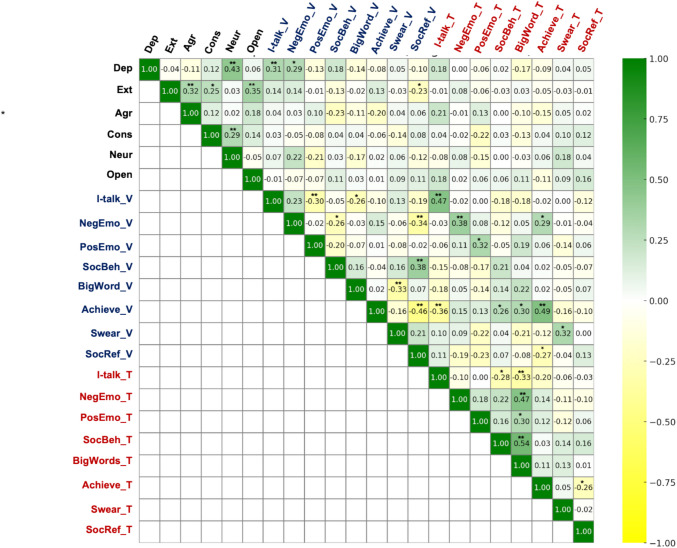
Pearson correlation heatmap of self-reported psychological variables and language variables *Note.* Figure shows Pearson correlations between self-reported psychological variables and diary-assessed language variables, with correlation values ranging from −1.00 (shown in yellow) to 1.00 (shown in green). Voice-assessed language variables are colored in blue, and text-entry-assessed language variables are colored in red. Self-report psychological variables are colored in black, and abbreviations are defined as follows: Dep = depression, Ext = extraversion, Agr = agreeableness, Cons = conscientiousness, and Neur = neuroticism. *indicates Pearson correlation with value *p* <.05, ** indicates Pearson correlation with value *p* <.01, *** indicates Pearson correlation with value *p* <.001. See Supplementary Document Table S5 for means, standard deviations, and confidence intervals (https://osf.io/jbwu4)

Correlations are reported as a correlation heatmap in Fig. 6. See Supplementary Document Tables S5–S7 (https://osf.io/jbwu4) for further reporting, including confidence intervals. Asymptotic *z* tests using Fisher’s *r*-to-*z* transformation to test differences in correlation coefficient values are reported in Table 6.

**Table 6:** Asymptotic *z* tests of language correlations derived from voice versus text-entry diaries

Association	Text-entry *r*	Voice *r*	*z* score	*p*
Depression ~ I-talk	0.181	0.306**	1.062	0.288
Depression ~ Neg emo	0.000	0.287**	2.224	0.026
Depression ~ Neg tone	−0.081	0.284*	2.738	0.006
Extraversion ~ Pos emo	−0.060	−0.013	0.336	0.736
Extraversion ~ Soc beh	−0.026	−0.129	−0.690	0.490
Extraversion ~ Soc refs	−0.009	−0.231*	−1.433	0.151
Openness ~ Big words	0.106	0.027	−0.532	0.594
Conscientiousness ~ Achieve	0.040	−0.056	−0.382	0.702
Conscientiousness ~ Neg emo	−0.018	−0.047	−0.219	0.826
Agreeableness ~ Neg emo	−0.009	0.029	0.286	0.774
Agreeableness ~ Swear	0.049	0.038	−0.079	0.936
Agreeableness ~ Soc refs	0.023	0.063	0.254	0.799
Agreeableness ~ Pos emo	0.132	0.101	−0.224	0.823
Neuroticism ~ Neg emo	0.081	0.220	1.067	0.286
Neuroticism ~ Anxiety	−0.074	0.315**	1.732	0.083
Neuroticism ~ Anger	0.010	0.050	0.257	0.796
Neuroticism ~ I-talk	−0.085	0.073	1.292	0.196

*indicates Pearson correlation value with *p* <.05, **indicates Pearson correlation value with *p* <.001

#### Depression 

For language assessed via voice diaries, significant positive associations were observed between depression and first-person pronouns, *r*(71) = 0.31, *p* = 0.008, 95% CI [0.08, 0.50], negative emotion words, *r*(71) = 0.29, *p* = 0.014, 95% CI [0.06, 0.48], and negative tone, *r*(71) = 0.28, *p = *0.015, 95% CI [0.06, 0.48]. These associations were all nonsignificant for linguistic features assessed through text-entry diaries (Fig. 6). A Benjamini–Hochberg correction accounting for 36 total comparisons between self-reported psychological variables and language variables resulted in corrected significance thresholds of *p* < 0.001 to *p* = 0.007, depending on the test’s ranking; no previously significant associations retained their significance at this adjusted threshold. As shown in Table 6, asymptotic *z* tests found a significant difference in correlation coefficient values between voice diary and text-entry diary methods in negative emotion words and negative emotional tone.

#### Personality 

Associations between personality domains and linguistic features were largely nonsignificant across language collected using both voice diary and text-entry diary methods (Fig. 6). Significant positive associations were observed between neuroticism and Fabla-assessed anxiety language, *r*(71) = 0.31, *p = *0.007, 95% CI [0.09, 0.51], and a positive association was observed between neuroticism and Fabla-assessed negative emotion overall, although this association did not reach the threshold for statistical significance, *r*(71) = 0.22, *p = *0.061, 95% CI [−0.01, 0.43]. There was a significant negative association between extraversion and social referents in the voice diary condition, *r*(71) = −0.23, *p* = 0.048, 95% CI [−0.24, 0.22]. No associations between personality domains and linguistic features assessed through text-entry diary were significant. The correlations did not differ in magnitude across methods.

## Discussion

Fabla is a novel ecological assessment app that collects participants’ spoken responses to researcher questions. This study put the app to its first empirical test in comparison with text-entry qualitative responding using a widely used survey platform. Participants rated the Fabla app as highly usable and acceptable, and nearly three quarters of participants indicated a preference for speaking their diary entries instead of providing a text-entry response. Spoken diaries yielded greater language volume per entry by a large margin—an average of twice as many words per day, and a 49% increase in responses with at least 150 words. Adherence in number of entries for this counterbalanced study was strongly driven by which diary method participants received first, and similar rates of adherence were observed between methods. These results provide evidence that a voice-response format for EMA increases the volume of data obtained in an acceptable—and often preferred— alternative to traditional text-entry responding.

The finding that voice diaries elicited double the volume of daily language data raises the question of how these data compare in terms of linguistic features that are widely studied. Overall, this study observed similar patterns of word-count-adjusted rates of language categories within voice and text-entry diaries, indicating that linguistic features of long-standing interest to daily diary and EMA studies can be readily collected through speech. Most linguistic features were moderately correlated between methods. For example, the Pearson correlation coefficient between I-talk in voice diaries and I-talk in text-entry diaries was 0.47, and the correlation between methods for negative emotion words was 0.38. This is suggestive of the strong role that participant traits may play in language production: our within-person analyses indicate that overall, participants in this study used similar patterns of language regardless of whether they were talking or typing.

However, ANOVA tests also identified systematic mean differences in the presence of strong correlations for several language categories, indicating that significant differences in how participants used language emerged when talking compared to when typing. Text-entry diaries elicited higher rates of words seven letters or longer, perhaps reflecting one participant’s observation that text-entry response allowed them to make entries “more professional” in contrast to the un-editable and raw quality of voice recordings. Other differences included that participants used higher rates of present-focused and cognitive process language when speaking and higher rates of past-focused language and first-person pronouns when writing. Effect sizes for observed differences tended to be medium to large (Richardson, 2011), although in proportional terms, these differences were all less than 2% of total word count (e.g., 11.60% utilization of first-person singular pronouns in text-entry diaries compared to 10.00% in voice diaries). Some statistically significant differences were observed for means within 1% of one another (e.g., for we-talk and she/he language). Whether such proportionally small differences reliably reflect variations in psychological symptoms or processes remains an area for further research. Given that large language models are commonly developed from text-based language samples, these findings indicate that further research into systematic differences in how people use language when talking compared to when typing is also warranted. If there are systematic differences, and if these differences track with real-world outcomes, this calls into question whether text-based language samples are appropriate for training models applied to speech-based samples.

Although language markers of depression (first-person singular pronouns, negative affect language, and negative tone) were observed at higher word-count-adjusted rates in text-entry diaries than voice diaries, these language features were significantly associated with depression symptoms only for voice diaries. In prior literature, associations between these language markers and depression have been replicated in both textual and spoken language samples (Holtzman, 2017; Koops et al., 2023; Tølbøll, 2019). Although our results require replication in larger and more diverse samples, this finding suggests that voice assessments of these language features may be more strongly linked to depressive symptoms than text-based assessments. This may result from method variance, such that participants may produce linguistic features because of the written format and not because of psychological constructs that manifest in, for instance, first-person singular pronouns, and which share variance with depression. Associations with previously observed linguistic correlates of personality were almost entirely nonsignificant for both text-entry and voice diary methods, although language derived from voice diaries did replicate some known associations for the neuroticism personality domain. In the meta-analysis of LIWC-derived correlates of Big Five personality domains that guided the selection of associations tested here, Koutsoumpis et al. (2022) note the tendency toward very small effect sizes and significant funnel plot asymmetry indicating publication bias in these domains. Our null findings are reported comprehensively as an additional data point for the field with regard to conditions under which language correlates of personality can be identified.

## Limitations

This preliminary study of a novel assessment method had several limitations. Our sample size of *n* = 87 individuals (and *n* = 59 participants with complete data for our most restrictive analyses in RQ2) left this study underpowered to test meaningfully potential demographic and psychological covariates of language effects, leaving this an important direction for future research. Relatedly, associations observed between language and depression symptoms were no longer statistically significant after correcting for multiple comparisons, underscoring the importance of future confirmatory research with fewer planned tests. This study is also unable to examine the extent to which language use in diaries varies or is stable day-to-day, or how momentary affect (e.g., transient states like excitement or anger) may influence daily diary language production. Prior laboratory studies have demonstrated that mood inductions can alter participants'language during simulated behavioral tasks, such as providing employee performance feedback (Forgas & Tehani, 2005). Future research investigating the degree to which variability in speech features is accounted for by transient mood states will be critical to identifying the best use cases for voice-based assessments of mental health symptoms that are more stable in nature, such as depressive episodes.

Our study population of undergraduate students who participated for extra credit is most accurately characterized as a convenience sample, restricting the generalizability of these findings. This study partnered with three universities in differing regions of the United States to attain a more diverse sample of undergraduate students than would have been attained through one university alone. Nevertheless, the age range in this study was 18–24 years, and results attained here cannot be assumed to generalize to more age-diverse or educationally diverse community samples. Age-stratified research on voice-to-text technology has found that older participants are *more* likely to express a preference for speaking into their phones to complete health information tasks than younger adults because manual query burden is a greater concern for older individuals and privacy is a greater concern for younger individuals (Bonilla et al., 2022). It is unknown whether this preference extends to EMA and similar assessment technologies, although if it does, the effects on language volume identified here may be larger for older populations. Replication in larger and more diverse samples is necessary to test the generalizability of the present findings and lay the foundation for a “voice EMA” methodology.

Finally, this study cannot address the question of what role self-editing played in observed effects. Qualitative feedback indicated that participants described their experience of responding through the Fabla app as “raw” and “stream of consciousness.” However, the Fabla app allowed participants to delete and rerecord entries if they chose to, and to protect participant autonomy over the data shared with researchers, the app did not track when this occurred. This is somewhat analogous to typed responses, where researchers typically cannot determine whether participants edited their responses using the “backspace” key. However, an important difference is that editing a typed response can occur continuously while writing, whereas editing a spoken response in Fabla required deleting the entire recording and starting over from the beginning. Future research should explore whether—and how—participants opt to re-record spoken replies to researcher questions.

## Data-driven improvements to Fabla and voice EMA methodology

Findings from this study have implications for the implementation of voice-based EMA in future research. Although the majority of participants in this study reported a preference for speaking their daily diaries, one quarter of the sample preferred text-entry. Many participants who preferred text-entry cited lack of privacy at the time of receiving the app diary notification as the primary reason for this preference. This preference is further reflected in use context patterns: participants provided a greater proportion of voice-entry diaries from home and a smaller proportion of voice-entry diaries from public locations, compared to text-entry diaries.

Our team has been continuing to develop and improve the Fabla app since this initial study in response to researcher requests for novel features and feedback from participants in this research. In direct response to this specific concern, we have added an option to the recording screen that participants can select to type an entry instead. Researchers can choose to enable or disable this feature; for example, researchers who require an acoustic data stream may prefer to disable this option. For research designs that do not require spoken language samples, however, this change may help capture reports from participants that would otherwise be missing. This may be particularly valuable for studies using a fixed assessment schedule (e.g., a response window of 1 hour), in which participants may have less ability to select the environments in which they provide responses. Studies collecting spoken-entry EMA using tools other than Fabla may benefit from providing participants with an alternate (typed or written) option for responding when speaking is not possible or practical.

Additional features recently added to Fabla based on researcher and participant feedback include integrated speech-to-text transcription via Amazon Web Services (AWS) Transcribe, a HIPAA-eligible tool, and camera access for collection of video-recorded and picture-based responses.

## Future directions for Fabla and similar tools

This study suggests that providing participants with an app for securely responding to researcher questions out loud using their smartphones is a low-burden approach to ecological assessment that generates rich data. We anticipate promising future applications for spoken EMA methods, whether collected via Fabla or through other applications.

### Facilitating mixed-method EMA and intensive longitudinal qualitative research

 Mixed-method designs have widely recognized advantages over purely quantitative approaches (Östlund et al., 2011; Sandelowski, 2000). Despite this, intensive longitudinal research protocols are heavily and often exclusively quantitative, perhaps due to the time burden imposed on participants by text-entry qualitative responding. Results of this study suggest that offering participants the option to speak their responses may help mitigate these challenges, making it more feasible to infuse intensive longitudinal research with mixed-method questions. There are numerous methodological advantages to including open-ended questions that participants can respond to out loud in intensive longitudinal study designs. These include participants’ ability to raise issues that researchers may not have considered querying at the momentary or daily level, the ability to capture aspects of experience that standardized questions miss, and the increased cultural inclusivity of giving participants an unrestricted ability to interpret, frame, and describe their experiences (Wood et al., 2024). From an analytic perspective, incorporating longitudinal qualitative questions also adds contextual depth, illuminating the “why” and “how” behind observed quantitative patterns.

### Increasing the scale and accessibility of single-time-point qualitative data collection 

Although this study aimed to test a longitudinal application of the Fabla app, the collection of single-instance qualitative data is the most basic application of Fabla and other tools like it. For example, structured qualitative interview questions can be programmed into Fabla and administered just once, making it possible to collect interview-like data from an unlimited number of participants without requiring any interviewer time from the research team. There are important limitations inherent in conducting qualitative interviews without a live interviewer: the absence of a human conversation partner eliminates the possibility of follow-up probes and may reduce engagement from participants who feel disconnected from the research team. However, given that the researcher time required to complete qualitative interviews ranks among the most commonly cited reasons for omitting qualitative data collection from a study (Bryman, 2007), app-based qualitative data collection may offer a solution for studies that would otherwise forgo qualitative data gathering altogether. For interview questions that are particularly vulnerable to social desirability effects and demand characteristics, responding via an app rather than through a live interview could conceivably also reduce these effects. Mode of administration, including the presence or absence of a live interviewer, has been found to impact participants’ willingness to disclose sensitive information in prior research (Levin-Aspenson & Watson, 2018).

### Identification of speech-based phenotypes and integration into measurement-based care

 This study focused on adherence, usability, acceptability, and concurrent validity of linguistic features as essential early steps in validating the Fabla app and advancing the development of spoken EMA methodology. However, speech data are multifaceted and contain numerous features relevant to clinical research not examined in this study. Paralinguistic features—acoustic properties such as tone, volume, and prosody—have demonstrated associations with several mental health symptoms and diagnoses, including depression (Hansen et al., 2022; Koops et al., 2023), anxiety (Albuquerque et al., 2021; McGinnis et al., 2019), post-traumatic stress disorder (Broek et al., 2010; Vergyri et al., 2015), schizophrenia (Chakraborty et al., 2018; Cohen et al., 2022), and neurocognitive disorders including Parkinson’s disease (Kodali et al., 2024; Ngo et al., 2022) and Alzheimer’s disease (de la Fuente Garcia et al., 2020; Haulcy & Glass, 2021). Additionally, speech recordings can be analyzed qualitatively through methods such as thematic analysis, enabling the identification of broader themes that may not emerge from linguistic or paralinguistic analysis. These include variables known to play an important moderating role in treatment response, such as recurrent cognitive patterns, beliefs, values, and lived experience of social relationships and social context.

Scientific interest in identifying “speech biomarkers” with predictive clinical validity has surged in recent years, driven by their potential advantages over biochemical markers and clinician-administered assessments (Insel, 2017; Koops et al., 2023; Robin et al., 2020). Speech sample collection is noninvasive, technically straightforward, and low-cost compared to extracting biochemical markers from saliva or sweat, and far more time-efficient than conducting individual clinical interviews. Speech biomarkers therefore offer a valuable potential resource for informing measurement-based care in mental health by offering a means for real-time, behavior-based, and scalable assessments. However, realizing this potential depends on the ability to translate speech assessments into valid and reliable predictive analytics. Machine learning models for speech classification that perform well on an initial sample of patients often fail when applied to patients who were not included in the training data, prompting recent calls for idiographic models that are fine-tuned to the person about whom predictions are being made (Hartnagel et al., 2024).

**Fig. 7:**
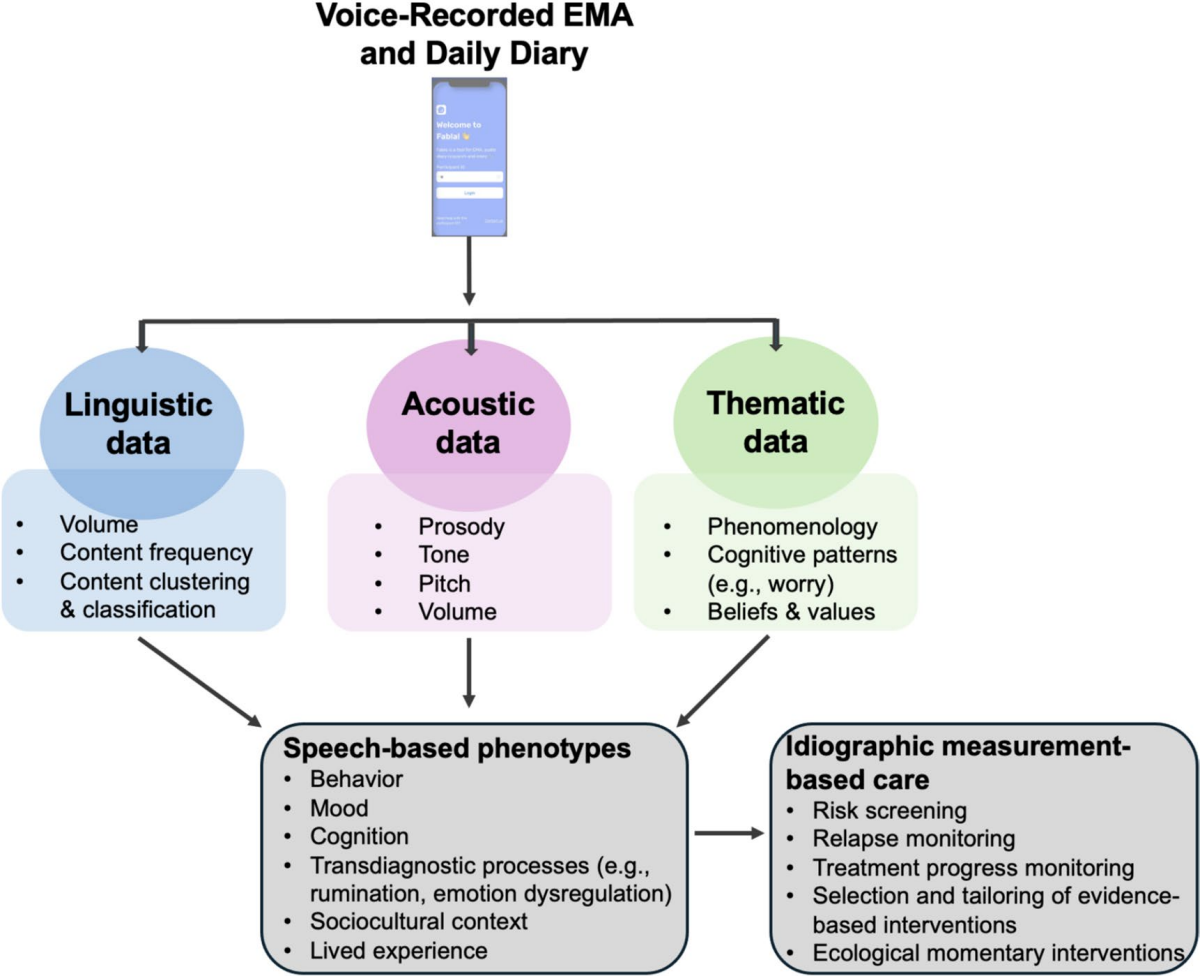
Integrating spoken EMA into measurement-based care. *Note.* Voice-based ecological assessment tools can inform measurement-based care through three broad categories of intensive longitudinal data. *Linguistic* features refer to the literal words people say, and are commonly analyzed using text analysis programs such as LIWC, VADER, and NLP-based models to generate data about the frequency and patterning of words that correlate with psychological attributes. *Acoustic* features comprise paralinguistic information extracted from speech such as prosody, tone, and volume, which also have demonstrated links to mental health outcomes (e.g., Ding & Zhang, 2023; Koops et al., 2023) *Thematic* features comprise the “bigger picture” expressed in voice recordings and include phenomenological data about lived social experience, recurrent cognitive patterns (e.g., worry, rumination, magical thinking), and overarching beliefs and values. These three sources of data can inform the nomothetic development of speech-based phenotypes. Models based on these phenotypes that are fine-tuned to the baseline of the individual being assessed can contribute to person-centered, real-time, and scalable measurement-based care.

Voice-based assessment tools like Fabla can play a crucial role in facilitating the large-scale, naturalistic data collection needed to develop and refine idiographic speech-based clinical tools. As these are developed, future generations of regulatory-compliant smartphone technologies may even include built-in and person-adjusted speech analytics that can signal improvements over the course of treatment or alert patients and healthcare providers of early warning signs of deterioration. The integration of longitudinally and naturalistically derived speech markers into measurement-based care has the potential to support personalized treatment planning, early detection of symptom escalation, and ongoing evaluation of therapeutic outcomes, ultimately enhancing the precision and responsiveness of mental health interventions (Fig. 7).

### Availability of Fabla and associated resources

As of the publication of this article, the Fabla app is maintained by these authors via the AppHatchery, the mobile app consulting service of Emory University and the Georgia Clinical and Translational Science Alliance. The authors have made Fabla available for use by research teams worldwide for the purpose of scientific investigations that aim to lead to generalizable knowledge. Interested research groups can email the corresponding author or visit www.apphatchery.org/projects/fabla. Reflecting our commitment to open science, data collection and analysis tools for the Fabla app will be posted at this URL and on the linked GitHub on an ongoing basis, as they are developed. The ongoing development and maintenance of Fabla is supported by the U.S. National Institutes of Health under grant UL1TR002378. The content is solely the responsibility of the authors and does not necessarily represent the official views of the U.S. National Institutes of Health.

## Data Availability

The data and syntax needed to reproduce the analyses reported in this article are available on the Open Science Framework page associated with this manuscript: https://osf.io/q258p/. Participant materials are also provided on this page. For participant privacy and in alignment with our study consent documents, raw daily diary entries are not provided on the Open Science Framework; however, the linguistic features (computed using LIWC-2022) derived from the raw diary entries are made available.
