# Correction: Fabla: A voice-based ecological assessment method for securely collecting spoken responses to researcher questions

**DOI:** 10.3758/s13428-025-02818-9

**Published:** 2025-09-04

**Authors:** Deanna M. Kaplan, Santiago J. Arconada Alvarez, Roman Palitsky, Hyoann Choi, Gari D. Clifford, Melese Crozier, Boadie W. Dunlop, George H. Grant, Morgan N. Greenleaf, Leslie M. Johnson, Jessica Maples-Keller, Holly F. Levin-Aspenson, Jennifer S. Mascaro, Ariel McDowall, Nicole S. Pozzo, Charles L. Raison, Ali John Zarrabi, Barbara O. Rothbaum, Wilbur A. Lam

**Affiliations:** 1 Department of Family and Preventive Medicine, Emory University School of Medicine, Atlanta, GA USA; 2 Department of Spiritual Health, Woodruff Health Sciences Center, Emory University, Atlanta, GA USA; 3 Emory University School of Medicine, Atlanta, GA USA; 4 Department of Psychiatry and Behavioral Sciences, Emory University, Atlanta, GA USA; 5 Wallace H. Coulter Department of Biomedical Engineering, Georgia Institute of Technology and Emory University, Atlanta, GA USA; 6 Department of Biomedical Informatics, Emory University School of Medicine, Atlanta, GA USA; 7 Department of Psychology, University of North Texas, Denton, TX USA; 8 Department of Psychiatry, School of Medicine and Public Health, University of Wisconsin-Madison, Madison, WI 53706 USA

**Correction: Behavior Research Methods (2025) 57:257**

10.3758/s13428-025-02777-1

The original online version of this article was revised: Figure 4 had an artifact included that obscured the data in the figure. The figure has been updated without the artifact.

